# Computer vision-based approach to detect fatigue driving and face mask for edge computing device

**DOI:** 10.1016/j.heliyon.2022.e11204

**Published:** 2022-10-20

**Authors:** Ashiqur Rahman, Mamun Bin Harun Hriday, Riasat Khan

**Affiliations:** Department of Electrical and Computer Engineering, North South University, Dhaka, Bangladesh

**Keywords:** Convolutional neural network, Drowsiness detection, Embedded system, Eye aspect ratio, Eye tracking, Fatigue, Nvidia Jetson Nano, Percentage of eye closure

## Abstract

The fatality of road accidents in this era is alarming. According to WHO, approximately 1.30 million people die each year in road accidents. Road accidents result in significant socioeconomic losses for people, their families, and the country. The integration of modern technologies into automobiles can help to reduce the number of people killed or injured in road accidents. Most of the study and police reports claim that fatigued driving is one of the deadliest factors behind many road accidents. This paper presents a complete embedded system to detect fatigue driving using deep learning, computer vision, and heart rate monitoring with Nvidia Jetson Nano developer kit, Arduino Uno, and AD8232 heart rate module. The proposed system can monitor the driver's real-time situations, then analyze the situation to detect any fatigue conditions and act accordingly. The onboard camera module constantly monitors the driver. The frames are retrieved and analyzed by the core system that uses deep learning and computer vision techniques to verify the situation with Nvidia Jetson Nano. The driver's states are identified using eye and mouth localization approaches from 68 distinct facial landmarks. Experimentally driven threshold data is employed to classify the states. The onboard heart rate module constantly measures the heart rates and detects any fluctuation in BPM related to the drowsiness. This system uses a convolutional neural network-based deep learning framework to include additional face mask detection to cope with the current pandemic situation. The heart rate module works parallelly where the other modules work in a conditional sequential manner to ensure uninterrupted detection. It will detect any sign of drowsiness in real-time and generate the alarm. The system successfully passed the initial lab tests and some actual situation experiments with 97.44% accuracy in fatigue detection and 97.90% accuracy in face mask identification. The automatic device was able to analyze different situations of drivers (different distances of driver from the camera, various lighting conditions, wearing eyeglasses, oblique projection) more precisely and generate an alarm before the accident happened.

## 1 Introduction

**Figure 1:**
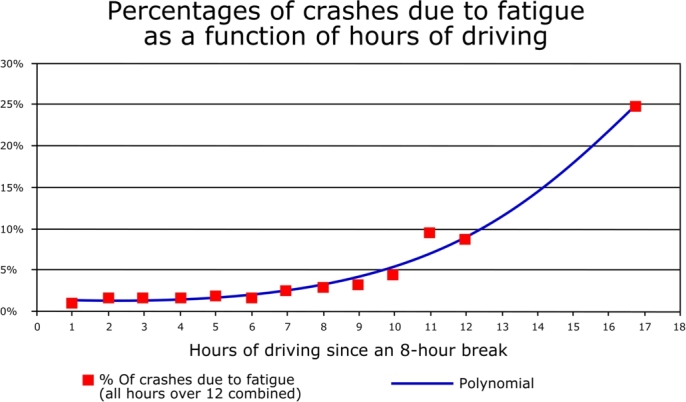
A graph shows how fatigue is related to vehicle crashes.

According to statistics, driver fatigue plays a critical part in traffic accidents, which have become a global concern in recent years [36]. Human factors in vehicle collisions or road accidents include drivers and other road users or roadside objects contributing to a crash or mishap [37]. Examples include the behavior of drivers, visual and auditory clarity, quick decision-making ability, and quick response speed. According to a study based on British and American collision statistics, driver error, tiredness, alcohol consumption, and other human variables account for 93 percent of all collisions [1]. So, human factors are the core reasons behind many traffic accidents. Rather than drugs or alcohol, fatigue is four times more likely to cause impairment [2]. Fatigue means feeling abnormally sleepy, decreasing the typical efficiency of cognitive functions [38]. Drowsy people may fall asleep in inappropriate situations or inconvenient times and make their surrounding situation highly critical. So, analyzing the problem statement, it is necessary to integrate modern technology to control road accidents due to drivers' drowsiness. Fig. 1 depicts how weariness is linked to traffic accidents, demonstrating the necessity for a drowsiness monitoring system to inform drivers if they become drowsy. When driving for more than 8 or 9 hours in a row, the number of accidents climbs rapidly.

The increasing death rates due to fatigued driving are quite alarming worldwide [39]. The death tolls in South Asian countries such as Bangladesh are getting terrible. Several simulation-based attempts to identify real-time fatigue in drivers have been constructed and modeled by various developers worldwide in recent years. These systems are later associated with high-end vehicles such as Volvo, Tesla, Mercedes-Benz, etc. However, the proposed schemes are out of reach for the middle class, particularly in South Asia. Expensive equipment is difficult to integrate into automobiles in Bangladesh, and high-maintenance gadgets are not user-friendly. This study aims to develop a low-cost, fully integrated fatigue and face mask detection system. The system uses computer vision and heart rate monitoring to identify fatigue and face masks, with the demands of South Asian transportation networks in mind.

Researchers have taken the following measures to determine the drowsiness of drivers, such as (i) vehicle-based measures, (ii) behavioral measures, and (iii) physiological measures [40]. The vehicle-based measures include vehicle deviation and position, speed, acceleration, and steering wheel movement. The behavioral measures involve eye movement, head poses, facial expressions like a yawn, eye blink, and others captured by the camera. Finally, Electrocardiograms (ECG), Electromyography (EMG), and Electroencephalograms (EEG) are examples of physiological measurements [3].

The aim of this work is to develop a system that could deal with fatigue driving using multiple input data sources. This integrated system is user-friendly, cost-efficient, and easy to use. This system takes input data from both the onboard camera and heart rate monitor and works independently to ensure system reliability in some extended challenging situations. It requires low power to run as it can operate with traditional batteries or regular power-banks or even with a DC-to-AC power car inverter to never run out of energy. Modern approaches like EAR (Eye Aspect Ratio) and mouth openness ratio, model-independent, are utilized to identify tiredness in this research. The point of interest vanishes in a critical drowsy state in the circular eyeball detection approach, which can sometimes be corrupted [41]. However, in the employed EAR and mouth openness approach, the interest points are always in the zone and do the work even when the individuals completely close their eyes. This system works with any transparent or semi-transparent eyeglasses. Even when wearing an eye lens, this system still works typically. The proposed automatic driver drowsiness identification device detects 68 different facial points to detect eye and mouth precisely. In the tests, the maximum distance to capture and analyze the state was 4-5 feet, which is undoubtedly sufficient for any vehicle. So, with this system, drivers can drive with complete comfort without noticing the camera. This is the first system that integrates EAR, mouth openness techniques, and heart rate monitoring for simultaneous drowsiness detection and face mask identification in this new situation during the coronavirus pandemic. The system employs a convolutional neural network model to recognize the face mask. The face mask detection model is accurate enough to detect any face mask, which has been trained with an open-source face mask detection dataset. The integration of EAR, mouth openness and heart rate monitoring reduced the chance of false results and complete system failure. Both systems are connected with a central alarm system yet independent of their work. It also gives the users complete control of comfort and safe drive. Drivers can use both modules simultaneously, as they can use anyone for their convenience. The system will still detect the drowsy states with almost the same accuracy.

The proposed system implemented in this work initially checks the face mask on the driver. When a face mask is worn, the system recognizes it and identifies drowsiness using the heart rate module. The computer vision module begins detecting when the driver removes the face mask, and the system receives the required frames to recognize the EAR and yawn. If either of these two modules detects any signs of drowsiness, the system generates an alarm immediately to keep the driver awake. The system can also ignore regular eye blinks and mouth openness when speaking specific words, e.g., “wow,” which causes the mouth to open as if yawning.

This paper implements an automatic fatigue driving and face mask detection system to warn drowsy drivers about potential road accidents. The following are the significant contributions to this work:

• An autonomous fatigue driving detection system has been developed utilizing deep learning, computer vision and heartbeat observation. Eye and mouth localization through computer vision is utilized to detect the states. The necessary values in this detection method are computed using a 68-point facial landmarks localization.
• An automatic face mask detection system is added using a deep convolutional neural network model.
• Diverse real-life circumstances are considered to detect driver drowsiness, e.g., different distances of driver from the camera, various lighting conditions, wearing eyeglasses, oblique projection, wrong masks wearing, etc.
• A comprehensive experiment is performed to verify the threshold EAR values before using them in the core system.
• The Nvidia Jetson Nano developer kit is used in the fatigue driving and face mask detection processes.
• The AD8232 heart-rate sensor is used with an Arduino Uno microcontroller to measure the heartbeat change during drowsy driving.
• The performance of this embedded system is evaluated in terms of accuracy from the achieved real-time experimental data. Finally, the features and performance of this paper are compared to other existing works.

To the best of our knowledge, the computer vision and heart-rate based fatigue driving and face mask detection approaches are implemented on an advanced embedded device, i.e., Nvidia Jetson Nano developer kit, for the first time in this work.

Some of the recent works on automatic driver fatigue detection are briefly described in Section 2. In Section 3, the required components and methods to implement the proposed system are described. In Section 4, real-time experiments results and evaluation of the proposed automatic device are discussed. Finally, Section 5 comprises the conclusion and future works.

## 2 Related works

Automatic driver drowsiness detection to prevent road accidents and fatal deaths has been studied extensively in recent times. The automatic techniques to detect driver drowsiness involve complex image processing, computer vision techniques, and deep learning approaches. For instance, in [4] and [5], SWM (steering wheel movement) is introduced as a vehicular measure to detect drowsiness using an angle sensor. It is not always possible to consider the geometric characteristics of the road. In developing countries, the roads are too narrow and consist of potholes in most cases. So the steering wheel movement varies in terms of the road condition. This technology will not be reliable enough to detect drivers' drowsiness in streets with cracked surfaces. Because of these road conditions, the drivers may have to drive differently, and the driver's steering behavior may change according to these unavoidable factors. If SWM technology is used to measure the drowsiness of a particular driver who is driving on such damaged and narrow roads, the vehicle's kinetic energy fails to remain constant, and the steering wheel movement changes without being drowsy. In [6], the authors proposed a face detection and eye localization method using symmetry for automatic drowsiness detection. They worked with eye localization and direct facial expression as trained data symmetry. Initially, this work used facial zone identification for the interest zone and then used eye localization to find out the current state of the eyes. Next, the circular localization system has been utilized for eye state detection, which identifies the eyeball of each eye. As long as the eyeball is detected, the system considers the driver is awake and working in an active driving state. Conversely, when the system fails to detect the eyeball but can detect the face in the frame, the system assumes that the driver is in a drowsy state and is not in active driving condition.

In [8], the authors proposed a localized edge detection of required zones from the face. The eye and mouth are localized using the circular edge of localizing zones. The closed-eye and open-mouth ideas are used to detect drowsy states. Consequently, face capture extracts the interest zones. Then they used the support vector machine technique, also known as the SVM technique, to develop the mechanism for detecting faces. The facial zone was then divided into reduced interest zones, including the mouth and both eyes. The proposed system recognizes microsleep periods (short sleep of approximately 2-6 seconds) to identify real-time conditions. Edge finding technology-based on Circular Hough Transform was utilized to detect the circular interest zone in the eye and mouth detection. Finally, the authors separated the state of their local area into several states. The states of mouth were divided into closed, slightly open, and widely available conditions to detect the desired drowsiness. B. Mandal and his team used a supervised spectral regression (SR) learning approach to develop an automatic fatigue detection system model [9]. The authors created the system specifically for bus drivers, facing a unique job challenge in oblique visual projection by the dome camera module. The authors resolved the issue using a fusion approach that combines adaptive quadrature (adaptive integration) and the most widely used drowsiness metric, “PERCLOS.” The authors implemented two sets of eye detection systems for more dependable and accurate eye detection using the dome camera mounted on buses, i.e., OpenCV eye detection and I2R eye detection. They developed their dataset with 23 volunteers, resulting in nearly 1,00,000 frames from over 230 minutes of video. Finally, the authors attained 97.1% accuracy for the left eye and 96.7% for the right eye on the train set, compared to 87.6% and 83.9% accuracies on the test set for the left and right eyes, respectively.

In [16], a 3D-deep convolutional neural network has been used to build an automatic driver drowsiness detection system. The authors used four different models, viz., Spatio-temporal representation learning, scene condition understanding, feature fusion, and drowsiness detection. Their proposed model can work the same at different times. Using the feature fusion method based on the tensor product approach, they retrieved the Spatio-temporal representation and combined it with the vectors representing the scene understanding findings. Based on current breakthroughs in computer vision disciplines, these challenges are efficiently simulated using 3D-DCNN and fully connected neural networks. The NTHU drowsy driver database has been utilized to validate the performance of the proposed technique. Finally, this work accomplished 76.2% overall accuracy for the driver sleepiness detection. In [17], W. Zhang et al. built technology for non-intrusive drowsiness recognition based on computer vision technology. The authors used eye-tracking, image processing, the 7-point Stanford sleepiness, and the 9-point Karolinska sleepiness scale. They have used Fisher's linear discriminant to combine the indices and statistics. The tests gave 225 samples of level 0 (awake), 181 and 158 samples of levels 1 and 2, indicating drowsy and very drowsy conditions, respectively. They have tested with six participants in a high-fidelity driving simulator and finally attained driver drowsiness recognition accuracy greater than 86%.

Some of the researchers implemented the detection approach to smartphone applications. In [11], Y. Ed-Doughmi et al. used a recurrent neural network (RNN) to detect fatigued driving. This paper used the NTHU-DDD video dataset in RNN model training. The data samples were extracted from the video clips of different driving states. The entire dataset has 849 video clips of about 7 seconds each. The proposed multilayer architecture has six convolutional layers, four max-pooling layers, one flatten layer, and two fully connected layers. This system uses a smartphone application connected to a web server to alert trusted contacts. Finally, the proposed system provides 92% validation accuracy in fatigue driving detection employing the conv3d 3D RNN approach. In [14], A. Dasgupta and his colleagues used the percentage of eye closure (PERCLOS) method to develop a smartphone-based fatigue detection system. The system uses the PERCLOS metric to determine the states of the drivers. After violating the PERCLOS threshold, the system measures the voice-to-unvoiced ratio through the smartphone's microphone. The system demands an active touch response from the driver to satisfy the active state. If no response from the driver is recorded, the system generates the alarm and sends an SMS to the trusted contacts. The authors achieved a 93.33% fatigue state classification.

In some works, the automated fatigue detection task has been deployed in different embedded circuits, e.g., Arduino, Raspberry Pi, Nvidia Jetson, etc. For example, in [15], N. Karuppusamy and B. Kang used a recursive neural network (RNN), electroencephalography (EEG), and gyroscope to determine fatigue driving. They used an EEG sensor to capture the brainwaves related to fatigue states. The Arduino Uno microcontroller is used to capture the gyroscope data. Real-time frames of drivers are captured with a Basler Ace camera and used in the trained RNN model. All three of these methods are used in the final classification. Finally, the proposed system achieved 93.91% accuracy in identifying the fatigues of the drivers. In [13], A. K. Biswal et al. used video stream processing (VSP) to determine the fatigue states of drivers. The authors used the eye aspect ratio (EAR) concept to calculate the Euclidean eye distance. This system can convey location information to trusted individuals and issue alarms. The basic system was built using a Raspberry Pi 3 Model B. The proposed system achieved 97.1% drowsiness detection accuracy when tested on ten individuals with and without eyeglasses. In [7], the authors proposed an embedded system based on deep neural network model compression. This work divided the entire driver fatigue detection process into several models and then compressed them into usable form with a deep neural network. First, the entire face is divided into four models. In the detecting system, the facial boundary serves as an initial model. Then the authors made a model for the mouth only, which detects the yawns. They separated two eyes into two different models for the left and right eyes. Finally, the obtained data from the driver's drowsiness detection network were compressed to be implemented on an embedded system of Jetson TK1. The authors achieved the best overall performance in terms of accuracy, detection time, and frame rate with the VGG-16 based faster RCNN deep learning technique. In [12], the authors aim to develop a high-performance, robust fatigue driving detection system. They used a convolutional neural network (CNN) to train the proposed model. Next, they used the Nvidia Jetson Nano developer kit to implement the proposed system into a fully embedded device. The system uses the face detection network “LittleFace” and classifies detected faces into two states, i.e., small and large yaw angles. Then a specialized regression technique, the SDM algorithm, boosts the normal state classification by introducing an improved face alignment operation. EAR and MAR determine the fatigue state classification. Their proposed architecture has six convolutional layers and three max-pooling layers prior to the output layer. Lastly, the face detection network achieved 88.53% accuracy, and the proposed system achieved 89.55% overall accuracy.

This section shows that automatic driver drowsiness detection has been performed in many works by using advanced image processing and computer vision techniques. However, only a few researchers implemented this intuitive detection framework on edge computing devices. This circumstance has inspired us to deploy the automated driver fatigue recognition system into an embedded device, Jetson Nano.

## 3 The method and proposed system

The comprehensive and integrated system overview is presented in subsection 3.1 of this section, which gives a general understanding of the system. Subsection 3.2 introduces and describes the proposed system's software tools. These include general remarks on software tools, used versions, and specific applications. This system's hardware components, specifications, and specific uses are then presented in subsection 3.3. Subsection 3.4 is focused on the depiction of eye aspect ratio and yawn detection methods and architectures. Subsection 3.5 is focused on the face mask detection methods and convolutional neural network architecture on which the model is trained. This subsection also discusses the dataset and data preprocessing techniques utilized in this work. Subsection 3.6 presents the architecture and methods used in the heart rate detection module. Finally, subsection 3.7 summarizes the complete integration philosophy of the system.

### 3.1 System overview

**Figure 2:**
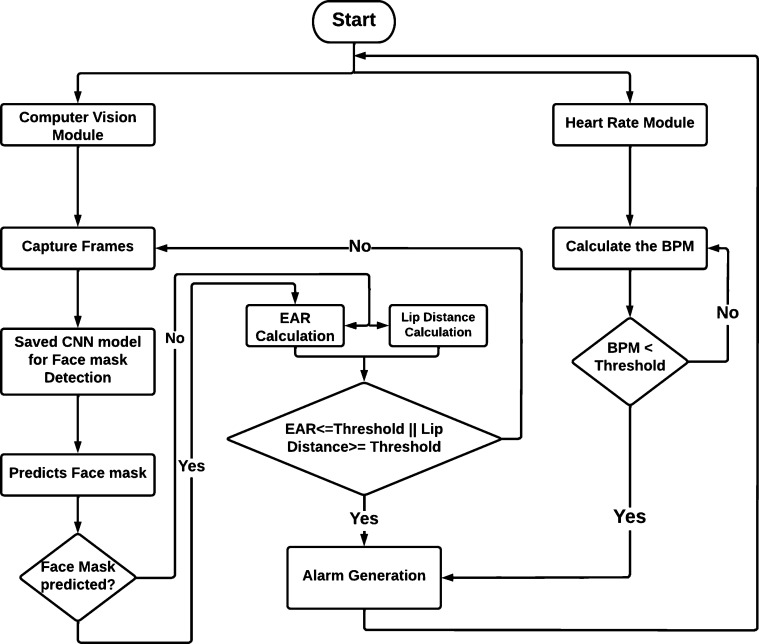
Integrated system flow chart.

After the initial boot-up, the proposed system loads the models and captures real-time frames through its onboard camera module. After getting the first frame from the real-time captured video, the system initiates the face mask detection process. Frames are sent to the trained model, and the model then analyzes the data and gives its prediction. After taking the predicted values, the system starts its logical procedures. On the other hand, the heart rate module monitors BPM values from the beginning. When the driver is wearing a face mask, the system detects frames through the camera module, and the heart rate module's results determine the driver's states. The heart rate module can decide the fatigue states independently. When the driver removes the face mask, the system gets the desired frames to detect 68 points on the driver's face. The system calculates the eye aspect ratio (EAR) and lip distance measurements from the specified eyes and lip points. The system classifies the driver's state by comparing the eye aspect ratio and lip distance to established threshold values. Finally, the system activates the alarm module when the driver shows drowsiness, generating a beep alarm with a 2 kHz signal frequency. The alarm will be active as long as the driver returns to the activate state and will stop after 5 seconds. This frequency range provides an environment that is not conducive to sleep, and the driver quickly returns to an active state. Fig. 2 shows the integrated system flow chart, which depicts the control flow of the system outlined in this paragraph.

**Figure 3:**
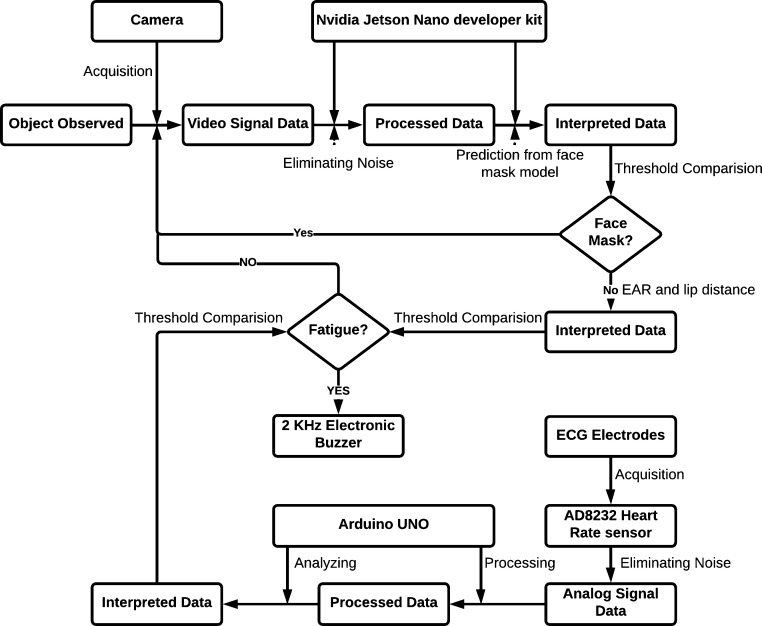
The framework of the proposed system.

The Nvidia Jetson Nano developer kit serves as the computer vision module's central piece of hardware. The heart rate module consists of an Arduino Uno as the system's primary controller and an AD8232 heart rate sensor to detect heartbeats. The Nvidia Jetson Nano program all the models and logic for the computer vision module outlined above. The Arduino Uno microcontroller is used to implement the heart rate module. Fig. 3 represents the integrated system framework.

The Nvidia Jetson Nano developer kit analyzes the video signal data and interprets it using saved models after retrieving it. The rationality is then checked, and decisions are made under the program. The Arduino Uno collects analog input from the sensor, processes it, and analyzes it before deciding. Until the user switches off the system, the entire system works iteratively.

### 3.2 Software tools

This integrated system's computer vision module uses a wide variety of software tools to analyze the driving states, which have been described below:

OpenCV [18] is an open-source computer vision and machine learning library. It is a common infrastructure in computer vision with more than 2500 optimized algorithms and lots of built-in functions. In this work, OpenCV Python version 4.5.1.48 is employed. This system makes extensive use of various powerful computer vision libraries. It was used to capture video frames, display output results, draw rectangles, circles, and other shapes, convert RGB to grayscale, and a variety of other tasks to ensure more accuracy in the system outcome.

Dlib [19] is a modern cross-platform C++ library known for data structure handling, machine learning, and image processing. Dlib version 19.22.0 is employed in this system for more accurate and consistent face detection and as the shape predictor, using a 68-point facial landmark detection model.

CMake [20] is an open-source tool for package testing and building software. In this work, CMake version 3.18.4.post1 organizes all the library packages installed to support the system.

SciPy [21] is a mathematical and scientific tool for the Python-based system. The most advanced scientific Python library is SciPy, which offers a practical module for computing the EAR and yawning in this paper. Scipy version 1.4.1 has been used, which provides various valuable functions for this system.

NumPy [22] is a Python-based open-source project that makes numerical computing easier. In terms of processing speed, NumPy outperforms other Python libraries. This system calculates the eye aspect ratio and lip distance using NumPy version 1.18.5. NumPy's inter-operability is its most essential feature.

Imutils package contains valuable functions for image processing. These functions are used in this system to process the frames. Imutils version 0.5.4 is incorporated in this system.

Keras is a Python interface to an open platform framework for neural networks. It is served as the interface for TensorFlow. Keras version 2.6.0 is used to train the face mask detection of this system.

Scikit-learn is a Python-based machine learning library that is simple and efficient for data analysis. It is developed to interact with the Python numerical and scientific libraries NumPy and SciPy. It includes SVMs, random forests, gradient boosting, k-means, and DBSCAN, among other classification, regression, and clustering techniques. It is used to build the face mask classifier of the proposed system.

The Arduino Integrated Development Environment (IDE) is a cross-platform application written in C and C++ for Windows, macOS, and Linux [23]. It is used to write and upload programs to Arduino-compatible boards. This tool is used to write the heart rate detection codes and upload the codes onto the board.

The PyCharm Community Edition 2020.3.5 is used as an IDE for Intel chipset machines run on Windows OS.

### 3.3 Hardware components

Nvidia Jetson [24] is a family of embedded computing boards. Nvidia's Tegra processor (or SoC) includes an ARM-64 architecture central processing unit in the Jetson TK1, TX1, and TX2 models (CPU). Jetson is a low-power system that helps machine learning and computer vision applications run faster. This system uses the Jetson Nano developer kit, which has 128 core Nvidia Maxwell GPU, Quad-core ARM A57 1.43GHz processor, 4 GB 64-bit LPDDR4 RAM with 25.6GBps transfer speed. All of these high-end specifications consume only 5 watts of power. As a result, this developer kit is ideal for AI applications. This serves as the computer vision module's central hardware unit.

Logitech C270 HD WEBCAM is used as the camera module. It captures real-time images in 720 pixels and 30 fps widescreen format. The module has a 55-degree field of view and auto light correction that helps the system get to a perfect frame. It has a firm mounting option and 5 feet long cable that helps place the camera module in suitable places. This camera module captures real-time frames and sends them to the core program in the Nvidia Jetson Nano developer kit.

Arduino Uno R3 is used in this work for the heart rate module's controller. Arduino Uno is a microcontroller board with 14 digital I/O pins and six analog inputs. It is widely used to control and run electronic and IoT devices globally.

AD-8232 [25] heart rate measurement kit is used to measure the heartbeat in this system. This heart rate measuring kit has an inbuilt noise reduction chip to detect and deduct the noises while measuring the heart rate.

Liquid-crystal displays (LCDs) are flat-panel displays that modulate light using liquid crystals and polarizers. A 16 × 2 LCD module displays the real-time heart rate.

A wired 2,000 Hz electronic buzzer alarm is used to generate alarms in this system. It is built with ABS (Acrylonitrile butadiene styrene) material, and the operable voltage is 5V. It generates a 2 kHz beep sound that was required by the system.

Baseus CRNBQ-01 in-car DC-to-AC power inverter is used to power the Nvidia Jetson Nano with the barrel jack input. This inverter uses DC power from the car and generates 5V/5A power suitable for the Nvidia Jetson Nano developer kit.

A 10,000 mAH power bank of Xiaomi is used to power the Arduino Uno. An Arduino Uno takes almost 500 mAH to run for 2 hours and 40 minutes [26]. So, a 10,000 mAH power bank can easily power the Arduino Uno for continuous 53 hours approximately.

Finally, the expected cost, size, and weight of the entire embedded system are shown in Table 1. All of the components were purchased in November 2021 from the local market in Dhaka, Bangladesh.

**Table 1:** The estimated cost, dimensions, and weight of the complete embedded system.

Component	Unit Price (in BDT)	Quantity	Total cost (in BDT)	Dimension (mm)	Weight (g)
Nvidia Jetson Nano	9,000	1	9,000	100 × 79 × 28 mm	249.5 g
Acrylic case	750	1	750	140 × 100 × 20 mm	100 g
Cooling fan	300	1	300	40 × 40 × 20 mm	40 g
Camera	2,200	1	2,200	73 × 32 × 67 mm	75 g
32GB UHS-1 SD card	1,100	1	1,100	–	–
Arduino Uno R3	500	1	500	68.6 × 53.4 mm	25 g
AD8232 sensor	800	1	800	28 × 37 × 3 mm	21 g
16 × 2 LCD	310	1	310	85 × 29.5 × 13.5 mm	35 g
Power bank	800	1	800	130 × 71 × 14.1 mm	228 g
Buzzer	15	3	45	–	1.6 g
Breadboard	55	1	55	165 × 53 × 10 mm	79 g
jumper wires	3	10	30	–	–
Subtotal = 15,890 BDT (185 USD)				Full system dimension ≈ 170 × 120 × 40 mm	Total weight ≈ 880 g

### 3.4 EAR (eye aspect ratio) and yawn detection

The EAR (eye aspect ratio) is a metric that determines eye openness or closeness. The proposed system uses the eye aspect ratio [27] to determine the active and fatigue or sleeping states. The EAR is computed using the Euclidean vertical and horizontal distance between the upper and lower eyelids. The EAR value of an open eye is higher than that of a closed eye. The system uses the percentage of eye closure (PERCLOS) [28] metric values to measure the alertness of drivers. PERCLOS is a fatigue detection metric that calculates eye closure rate over the pupil in real-time. It is established on slow eyelid closures or drops rather than regular eye blinks. This approach can ignore regular eye blinks and precisely detect sluggish eyelid closures or eyelid drops caused by fatigue driving using the PERCLOS measurement.

The complete EAR and yawn detection process consists of several steps. The system recognizes specific parts on a face to calculate the eye aspect ratio and lip distance. Before the system can detect the points, it first detects the faces. Face detection includes head and shoulder detection. Thus, the sequential process involves head and shoulder detection, face localization, eye and lip localization, point detection, and finally, the eye aspect ratio and lip distance calculation.

#### 3.4.1 Face detection

**Figure 4:**
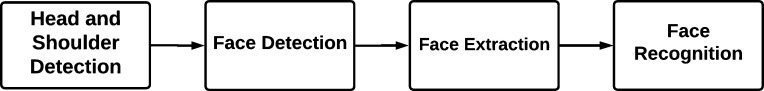
A generic face recognition system.

The first step of the proposed driver drowsiness identification system is to detect faces using the onboard camera module. Here, the camera works as an input device to capture the image of the driving person. The system can quickly detect faces from a live video stream using computer vision's most famous libraries, OpenCV and Dlib. Grabbing the frames from the video stream is a part of face extraction. Fig. 4 shows a generic face recognition process. This method starts from the head and shoulder detection then gradually to the face recognition. This process eliminates the background from the particular image and ignores any other foreign particle apart from the face region. Dlib and OpenCV both have frontal face detection capability. This system uses the Dlib library package of Python to detect the driver's face from the video stream input.

There are two different methods of detecting faces built into the Dlib library.

• Using HOG + Linear SVM face detector.
• Using Max margin (MMOD) CNN face detector.

**Figure 5:**
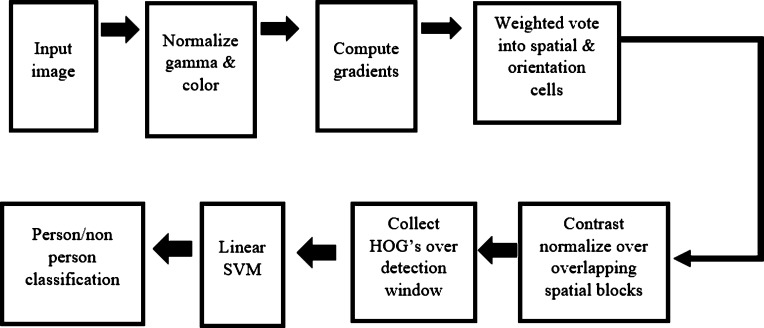
An overview of HOG algorithm for human detection [29].

In this work, the “HOG + Linear SVM face detector” has been used to give computationally accurate and efficient results. In [29], a human detection algorithm is presented that had efficient and excellent results. This detection process is represented by a dense grid of oriented gradients (HOG) histograms. This recognition method is powerful enough to classify human faces using a linear support vector machine (SVM) approach. Fig. 5 shows an overview of the detection process. In order to use the HOG + Linear SVM method for face detection with the help of Python, Dlib's function, “dlib.get_frontal_face_detector()” has been used in this work.

#### 3.4.2 Face localization

After successfully detecting the face, the system localizes the detected face. Face landmarks are detected as part of the face localization process. The detection of facial landmarks is a subset of picture shape predictors. The facial landmarks of the face region are listed below:

• Eyes
• Eyebrows
• Lips
• Nose
• Jawline

**Figure 6:**
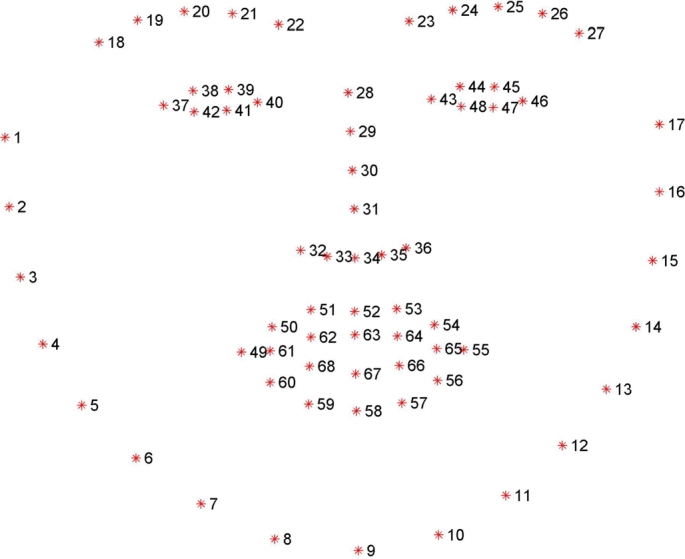
The 68 landmark facial landmarks coordinate from the iBUG-300W dataset [31].

After localizing these landmarks on the face region, one can easily extract the eye region by the particular landmarks located around the eyes using the shape predictor method. In order to assist the machine learning algorithm, the Dlib library was written in C++. The algorithm returns 68 distinct characteristic points for each individual in the provided frames using the “dlib.shape predictor” (“shape predictor 68 landmarks.dat”) function [30]. This article discusses how to get the frames using the “dlib.get_frontal_face_detector” function. In [31], a semi-automatic process was introduced for the landmark points, and that article presented and analyzed the results of different 300 faces in “The Wild Challenge (300-W)”. It was the first facial landmark challenge with existing methods compared. Fig. 6 shows the detected 68 individual landmark points with specific numbers. These landmark points are used to calculate the eye aspect ratio and the lip distance in this research.

#### 3.4.3 EAR and lip distance calculation

**Figure 7:**
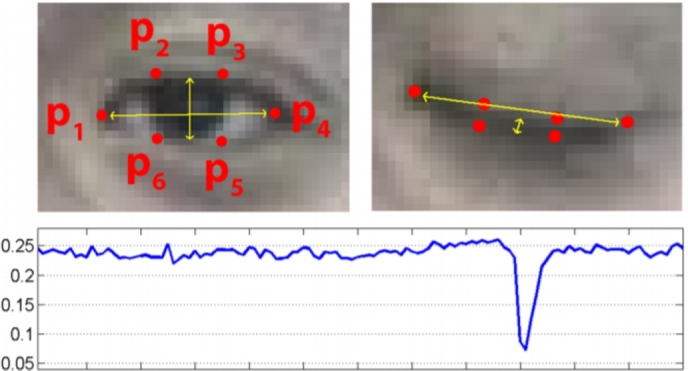
Open and closed eyes with automatically recognized landmarks with EAR drop graph.

The eye aspect ratio (EAR) is calculated with the help of vertical distance and horizontal distance of both left and right eye landmarks. Fig. 7 shows the landmark points of an eye. These six points are the generalized points to calculate the EAR. Point P_1_ and point P_4_ are generally considered as the horizontal points. Points P_2_, P_3_, P_5_ and P_6_, these four points are considered as vertical measurement points of an eye.

According to the points in Fig. 7, the vertical distance of an eye is calculated as:

(1) $$V_{W_{eye}}=||W_{P_{2}}-W_{P_{6}}||+||W_{P_{3}}-W_{P_{5}}||$$

In (1), the vertical and horizontal distances are expressed as V_W_eye__ and H_W_eye__, respectively, and W_P_i__ denotes the point's weight. Conversely, the horizontal distance of an eye can be found as:

(2) $$H_{W_{eye}}=||W_{P_{1}}-W_{P_{4}}||$$

Next, with the help of (1) and (2), the eye aspect ratio is calculated. The equation for EAR is represented by (3) as:

(3) $$EAR=\frac{V_{W_{eye}}}{2H_{W_{eye}}}$$

**Figure 8:**
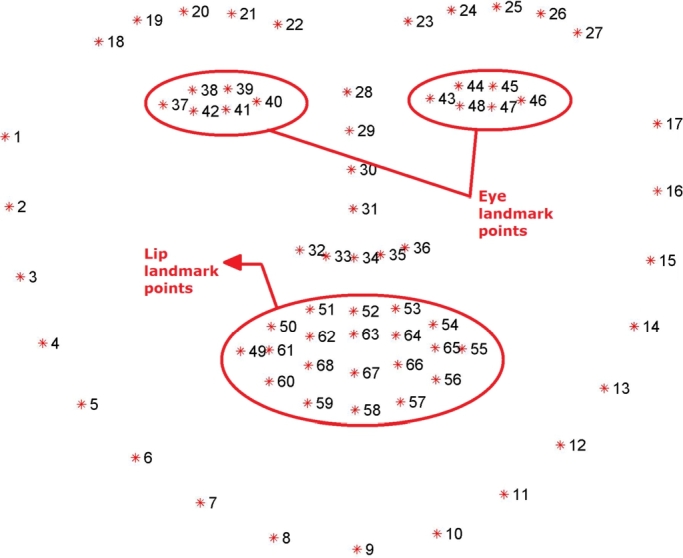
Eye landmarks and lip landmarks.

68 distinct points, as in Fig. 6, were determined with the face localization process. Eye and lip landmark points will be used to compute the EAR and lip distance. Fig. 8 shows the meticulous eye and lip landmarks from all 68 points.

The EAR is calculated separately for both left and right eyes. Each eye has six distinct landmark points from the face localization, as depicted in Fig. 8. For the left eye the weights of the distinct points are, [W_P_37__, W_P_38__, W_P_39__, W_P_40__, W_P_41__, W_P_42__]. For the right eye the corresponding points are, [W_P_43__, W_P_44__, W_P_45__, W_P_46__, W_P_47__, W_P_48__]. Consequently, the EAR equation for the left eye can be found as:

(4) $$EAR_{left}=\frac{\left||W_{P_{38}}-W_{P_{42}}||+||W_{P_{39}}-W_{P_{41}}|\right|}{2||W_{P_{37}}-W_{P_{40}}||}$$

The EAR equation for the right eye is,

(5) $$EAR_{right}=\frac{\left||W_{P_{44}}-W_{P_{48}}||+||W_{P_{45}}-W_{P_{47}}|\right|}{2||W_{P_{43}}-W_{P_{46}}||}$$

**Figure 9:**
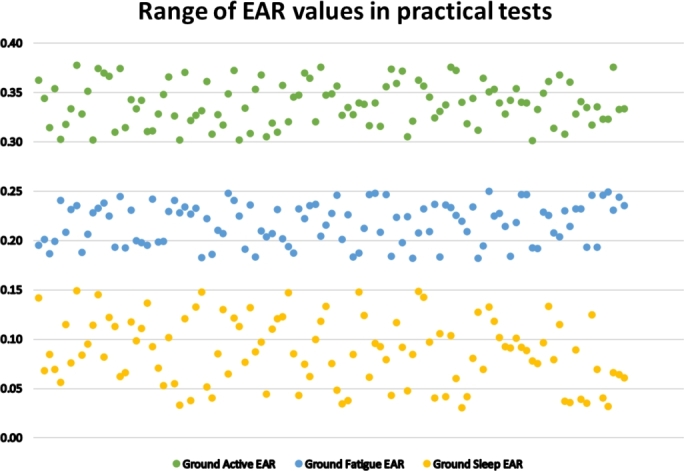
Range of EAR values in all three states.

In (4) and (5), $W_{P_{x}}$ denote corresponding weights of various eye landmark points. An extensive experiment in different lighting conditions has been carried out to determine the perfect threshold values of the EAR for the active, drowsy and sleepy states classification. Four male volunteers participated in this study, and their ages ranged from 23 to 29. Informed consent was obtained from the participants. The volunteers acted as if they were drowsy or sleeping to get the data. Fig. 9 demonstrates the experiment's EAR values' range for active, fatigue, and sleep states. The EAR values lie between 0.38-0.30, 0.255-0.18, and 0.155-0.03 for active, fatigue, and sleep states, respectively. The experiment produced a non-overlapping graph, which aids in determining the boundary for all three states' classification.

With the help of this graph, the threshold range (TH_range_) was determined. The range is,

(6) $$TH_{range}=\left\{ \begin{array}{cc} EAR\geq 0.28 & ;Active\\ 0.17<EAR\leq 0.27 & ;Fatigue\\ EAR\leq 0.17 & ;Sleep \end{array}\right.$$

Experimental results confirm that, on average, a regular eye blink takes 100-300 ms [10]. In this work, we used 1000 ms or 1 second of time rate to avoid normal eye blinks for EAR-based fatigue detection. The proposed system detects drowsiness when the EAR value, as expressed in (6), crosses the threshold for a continuous period of 1000 ms.

**Figure 10:**
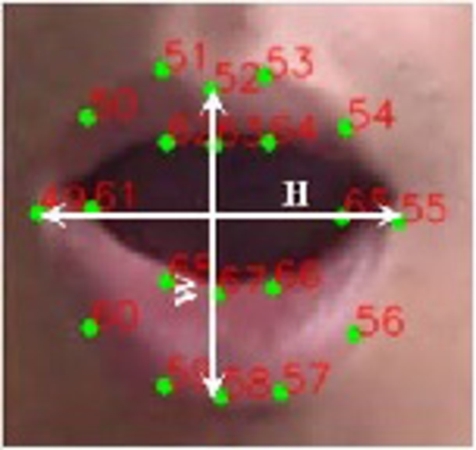
Top and lower lips landmark points.

Lip distance is used to classify the yawn, another form of fatigue sign. Interested points to calculate the lip distance are shown in Fig. 8 as “Lip landmark points.” The distance is calculated by subtracting the mean of the top lip weights from the mean of the lower lip weights. Fig. 10 shows the distinct points of both top and lower lips. It is worth mentioning that, the top lip and lower lip both contain eight distinct points each.

The mean of each lip is calculated by adding the weights of those points together. Equation (7) is used to sum up all of the top lip's interesting point weights.

(7) $$Top_{lipW}=\sum_{i=50}^{53}W_{P_{i}}+\sum_{i=61}^{64}W_{P_{i}}$$

Equation (8) is used for summing all the respective point weights of the lower lip.

(8) $$Lower_{lipW}=\sum_{i=56}^{59}W_{P_{i}}+\sum_{i=66}^{68}W_{P_{i}}$$

Next, the mean of top and lower lips is calculated as:

(9) $$Top_{lip\_mean}=\frac{Top_{lipW}}{8}$$

(10) $$Lower_{lip\_mean}=\frac{Lower_{lipW}}{8}$$

Finally, the lip distance is measured as:

(11) $$Lip_{distance}=||Top_{lip\_mean}-Lower_{lip\_mean}||$$

In (11), $Top_{lip\_mean}$ and $Lower_{lip\_mean}$ are obtained from (9) and (10), respectively.

### 3.5 Face mask detection

People use face masks to protect themselves after the new coronavirus disease outbreak. With everyone being asked to wear masks in public and crowded places, e.g., religious sites, public transport, airports, etc., there has been a need to detect the face mask automatically using artificial intelligence and computer vision techniques. The proposed system uses face mask detection as an additional feature to cope with this pandemic situation. The system uses a convolutional neural network (CNN) to train the face mask classifier. The proposed framework for the face mask classifier is separated into three modules, where the first module is “Data Preprocessing,” the second module is “Model Training,” and the third module is “Retrieval.” We analyzed the data and transformed it into a comprehensible form for the training module in data preprocessing. This classifier distinguishes between two classes, i.e., “with mask” and “without a mask.”

#### 3.5.1 Dataset

The open-source dataset used for training the model was acquired from Kaggle [32]. There are 5,988 images in the dataset, divided into two groups, “with mask” and “without a mask.” There are 2,994 photos in each class. Following a study of the data samples, we realized that there were enough samples for model training but not enough samples to identify occlusion. Then, to deal with occlusions, we added some more instances. We created the added samples on our own to deal with the occlusion. In this paper, two videos have been employed to extract frames for samples. We used an open-source video-to-JPG converter to extract the frames. This resulted in the total dataset consisting of 6,200 samples. Then we split the resulting dataset into train, test, and validation datasets. As a result, we end up with 4,464 training samples, 620 test samples, and 1116 validation images, all belonging to two different categories for final training.

#### 3.5.2 Data preprocessing

**Figure 11:**
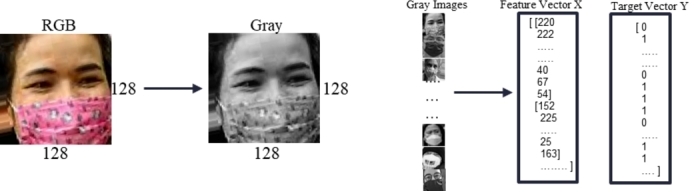
Illustration of data preprocessing steps.

The dataset we used to train our model had 6,200 images after the finalizing steps. In both classes, all of the samples were color images with 128×128 pixels in size. They had three channels, red, green, and blue. The dataset folder had two sub-folders to denote the two distinct classes. We first retrieved the subfolder categories and added numerical labels in this preprocessing module. The samples were then converted from color to grayscale images. The channel size was lowered from three to one, and the feature vector contained far fewer elements than color images. The feature vector for a single 128 × 128 color image has 49,152 elements. A single 128 × 128 grayscale image shrinks the feature vector element size from 49,152 to 16,384, which is three times less than the color image. The cv2.COLORBRG2GRAY function from OpenCV [18] transformed the color image to grayscale. We inserted the numerical values of gray images into NumPy [22] arrays. We added the equivalent target vector to our feature vector. Then finally saved the feature and target vectors for the “Model Training” module. Fig. 11 illustrates the visual representation of the utilized data preprocessing module.

#### 3.5.3 Model training

**Figure 12:**
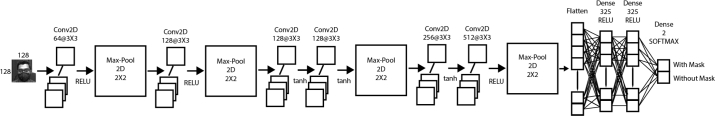
Architecture of the customized convolutional neural network.

In this work, a customized convolutional neural network with TensorFlow on the backend has been used for the model training of face mask detection. The convolutional and max-pooling layers can cope with random distortion and translation in the convolutional neural network. Additionally, the max-pooling layers can simplify the process by offering abstraction for the objects in the pooling. The input layer was a set of grayscale images, all resized in 128 × 128 pixels in one single channel. The input layer is then processed with six successive convolutional and four max-pooling layers. The first convolutional layer had 64 filters of size 3×3, and the max-pooling layer filter is of size 2 × 2. Then the applied second, third and fourth convolutional layers had 128 filters of size 3 × 3, and the max-pooling layer filter is 2 × 2. Finally, the sixth convolutional layer had 512 filters of size 3 × 3, and the max-pooling layer filter is of size 2 × 2. We incorporate Rectified Linear Unit (ReLU) and tanh as the activation functions between each convolutional and max pooling frameworks. After the fourth max-pooling layer, we introduced a layer in the network to flatten the output of the last max-pool layer and then passed it onto the two fully connected dense layers. These dense layers had 325 neurons. The activation function was the ReLU. Before the output layer, we had a final dense layer using the softmax activation function. We used Adam optimizer categorical loss and accuracy as the cross-entropy measure to train the final network during network training. The architecture of the employed customized CNN network is shown in Fig. 12. It is interesting to note that, the number of layers and the hyperparameters of the employed customized CNN framework have been selected using the validation subset of images of the dataset [32].

#### 3.5.4 Retrieval

**Figure 13:**
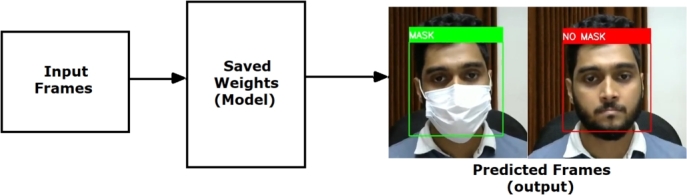
Illustration of the Retrieval Module.

In the retrieval module, we use the pre-trained deep neural network classifier that we trained and saved in the previous training module to compare and classify unseen frames provided by the user. This module captures the real-time video frames as the user input. Then it passes those frames to the loaded pre-trained classifier. Next, the pre-trained classifier subsequently classified each frame, delivering the results to this retrieval module. The numerical class values are then decoded, and the actual text label is added to each frame by this module. Finally, the labeled frame is shown in real-time as predicted results for each frame. The faces are detected in a rectangle box in the output frame, and the predictions are displayed in text forms. This module uses OpenCV [18] to capture video frames from the input device and display the output on the user's screen. Fig. 13 illustrates the abstraction of the retrieval module of the utilized sketch-based image retrieval system.

### 3.6 Heart rate detection

**Figure 14:**
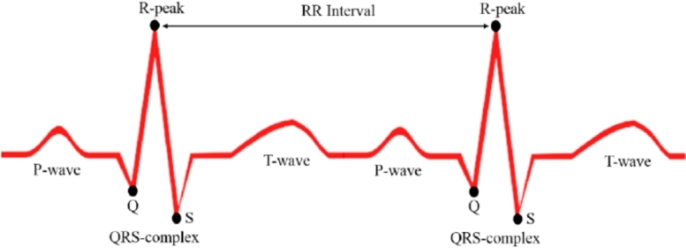
R peaks and RR interval between two successive R-waves.

The number of heartbeats in a minute is known as heart rate or heartbeat per minute (BPM) [42]. The heartbeat is often above a threshold value while awake and fully active. The type of exercise usually dictates a healthy person's heart rate [43]. In a fatigue or sleeping condition, a person's heart rate is lower than in an awake or active state [44]. This system detects fatigue by analyzing any drop in heartbeats below the threshold. HRV (heart rate variability) is observed from the interval of RR in an electrocardiogram (ECG). The ECG signal shows different results during stress and fatigue. Fig. 14 shows the R-peak of the QRC signal on the electrocardiogram. It also shows the RR interval, which is the interval between two successive R-waves.

The AD8232 heart rate sensor used in this work picks the time delay in milliseconds between two successive R-peaks, which means the time of a RR interval. The sensor eliminates noise from the signal and then detects the RR interval in milliseconds. The module controlling the core program in Arduino Uno then extracts the time signal and converts it into BPM, as shown in Fig. 3. The BPM can be calculated by dividing a minute with the RR interval in the same unit. The formula to calculate the BPM from the AD8232 heart rate sensor's signal is expressed as:

(12) $$BPM=\frac{60000}{Sensor_{signal\_in\_milliseconds}}$$

The heart rate sensor gives a signal in milliseconds. One minute is equal to 60,000 milliseconds. That is why the formula divides 60,000 by the analog signal from the sensor to calculate the real-time bpm in (12). This system uses a 500-millisecond delay interval to avoid unwanted noises between two R-peaks. So, for the proposed system with a 500 ms delay time, the heart rate equation becomes:

(13) $$BPM=\frac{30000}{Sensor_{signal\_in\_milliseconds}}$$

**Figure 15:**
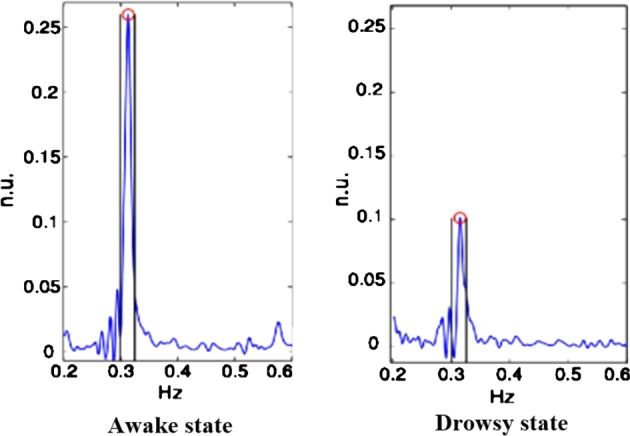
ECG graphs of active and fatigue states.

According to (13), this system requires 30,000 as the numerator of the formula to eliminate the 500-millisecond delay time. Fig. 15 demonstrates how the amplitude of the ECG graph dramatically decreases when recorded in a fatigue condition compared to the awake state.

There is a clear difference in heart rate between active and fatigued states. The data of the BPM study of male and female drivers' heart rates for active and fatigue states are shown in [34]. Table 2 shows the results of the BPM study on male and female drivers' heart rates.

**Table 2:** BPM analysis of male and female drivers.

States	Male	Female
Active	75 < BPM < 100	70 < BPM < 95
Fatigue	50 < BPM < 65	45 < BPM 63

As per Table 2, the highest BPM value in the fatigue state is 65. It does not overlap with the active state for either males and females. Hence, 65 BPM can be treated as the threshold value for classifying fatigue states. Accordingly, the threshold values for heart rate detection of active and fatigue states is determined as:

(14) $$TH_{Heart\_Rate}=\left\{ \begin{array}{cc} BPM\geq 68 & :Active\\ BPM\leq 67 & :Fatigue \end{array}\right.$$

Finally, this system uses 67 as the threshold value to get more flexibility, which has been expressed in (14).

### 3.7 Fusion

**Figure 16:**
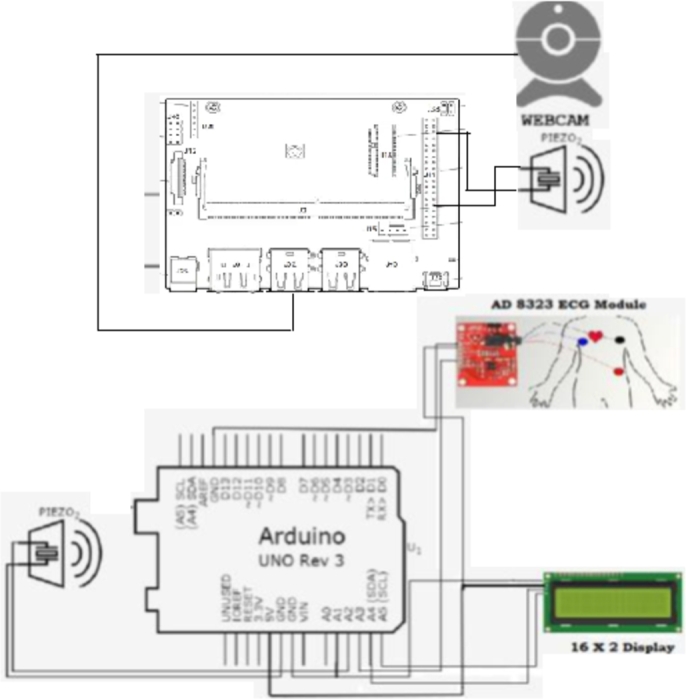
Schematic diagram of the complete embedded system.

The embedded system is comprised of sections 3.4, 3.5, and 3.6. The three sub-modules of this comprehensive embedded system are ear and yawn detection, face mask detection, and heart rate monitoring. The complete schematic diagram of the system is shown in Fig. 16.

**Figure 17:**
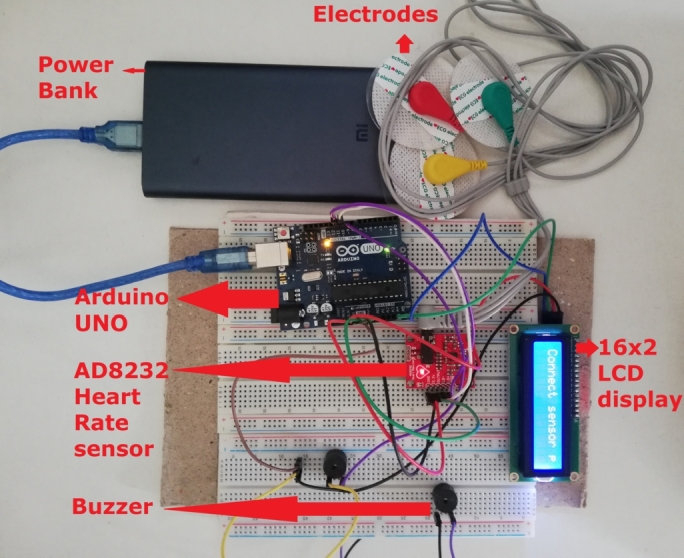
Complete heart rate module with all the components.

Fig. 17 shows the complete heart rate module with all hardware components. This module has individual power sources and is entirely independent of classifying the states.

**Figure 18:**
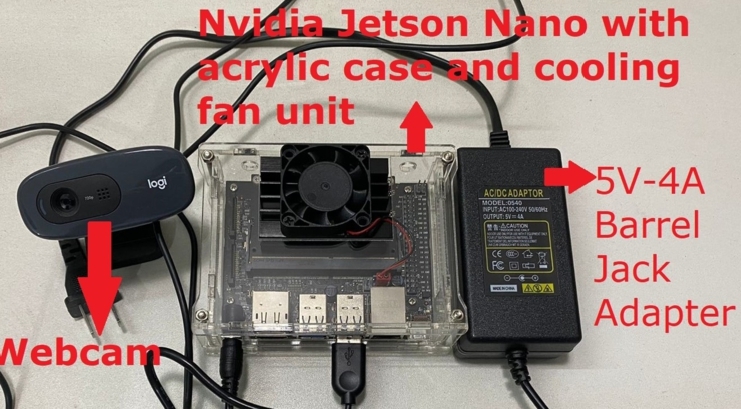
Entire computer vision module with all the components.

Fig. 18 shows the complete computer vision module of this system. It contains the Nvidia Jetson Nano developer kit with a cooling unit, one webcam, and one 5V-4A barrel jack adapter to ensure constant power to the system.

**Figure 19:**
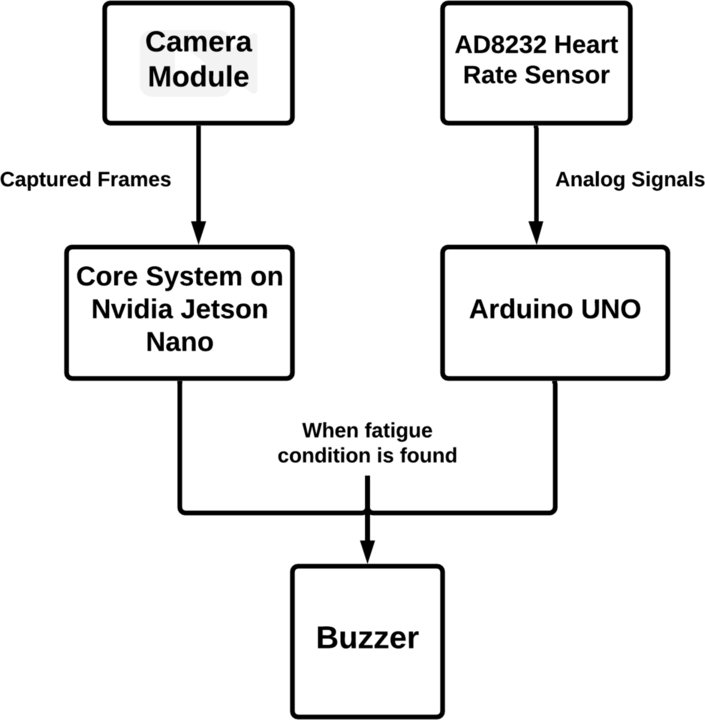
Embedded system's control flow.

Fig. 19 shows the general control flow of the embedded system. The actual control flows from system initialization to state detection and alarm generation are shown in this illustration.

**Figure 20:**
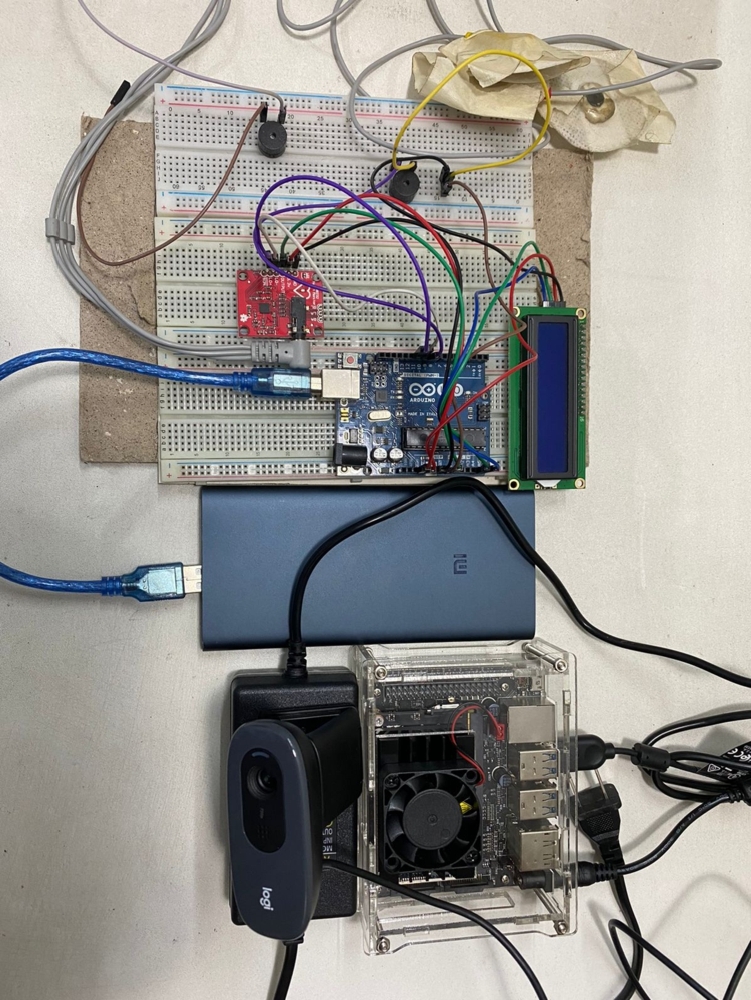
Complete embedded system.

Fig. 20 depicts the complete embedded system combining the heart rate sensor and computer vision module. The reason behind this fusion is to build a reliable, flexible, and durable system that can overcome some system failures. The system does not get the required frames to localize the face when the driver wears a face mask. At that period, the heart rate module can successfully classify any signs of fatigue from the BPM values. The heart rate module can run for approximately 53 hours without being recharged. That is considered to be enough driving time to charge the module. However, the computer vision module takes power from the car's battery using the DC-to-AC power inverter and can constantly operate until the vehicle is cruising. The Nvidia Jetson Nano is attached with a cooling fan to deal with the heat so that it does not stop. Finally, the proposed driver drowsiness detection system achieved more reliability and flexibility in many critical conditions with these fusions.

## 4 Experiments and evaluation

In this work, the authors focus on fatigue detection in drivers with the help of computer vision and heart rate monitoring technology. We have introduced face mask detection as an additional feature in this pandemic circumstance. This system's precision and reliability are essential since it helps to provide a safe driving environment. At every stage of the system's design approach, the authors focused on accuracy and reliability. The authors performed various experiments on the system after finalizing the design and completing the construction of the proposed automatic device. Several lab tests and real-world vehicle tests are among the experiments. Experiments were carried out on Windows machines with Intel chipset and the Nvidia Jetson Nano developer kit. The specifications of the desktop PC and Jetson Nano are illustrated in Table 3. Eight participants enrolled in the experiments, six of whom were male, and two were female. Experiments were conducted under various lighting conditions to assess the accuracy and reliability of the system in a variety of challenging scenarios. The system was tested at varying distances and projections, apart from diverse lighting conditions. The system was tested from two feet to 7 feet distance range, segmented into three parts. Segment I was a 2 ft. to 3 ft. distance, segment II was a 4 ft. to 5 ft. distance, and the last segment was a 6 ft. to 7 ft. distance. The ranges were picked from open-source car data. For every large-scale vehicle on the market, seven feet is considered sufficient distance from the dashboard and driving seat's position. The camera module was tested in an oblique projection perspective to guarantee placement anywhere on the dashboard. As a result, the system can classify a driver's state from a wide variety of distances, ranging from 2 feet to 7 feet, and efficiently classify states in oblique projection. The system was also tested with and without eyeglasses, ensuring that drivers who wear glasses will have no issues utilizing them. The experiments yielded convincing results, and the system is now ready to use.

**Table 3:** System specification used in the experiments.

	Desktop Computer	Nvidia Jetson Nano
CPU	Intel core i7-7700 with 3.60GHz	Quad-core ARM Cortex-A57 MPCore
GPU	GeForce GTX 1060 6GB GDDR5	Nvidia Maxwell architecture, 128 CUDA cores
RAM	16 GB	4 GB
OS	64-bit Windows 10 Pro	Linux4Tegra, based on Ubuntu 18.04
Storage	240GB M.2 SATA SSD 6Gb/s	32 GB UHS-1 card

The computer vision module's experiments and evaluation are discussed in Section 4.1. Section 4.2 discusses the experiments and evaluation of the heart rate module. Finally, the overall performance and system comparisons are evaluated in Section 4.3.

### 4.1 Experiments and evaluation of computer vision module

The computer vision module uses a convolutional neural network for face mask detection. The eye aspect ratio (EAR) and lip distance computation are applied for the driver's state classification. The eye aspect ratio and lip distance are generated using the 68 facial landmarks detected by the Dlib shape predictor. This module was tested on both Windows computers with Intel chipset and the Nvidia Jetson Nano developer kit. Table 3 shows the system specifications used in the experiments.

#### 4.1.1 Face mask experiments and evaluation

**Figure 21:**
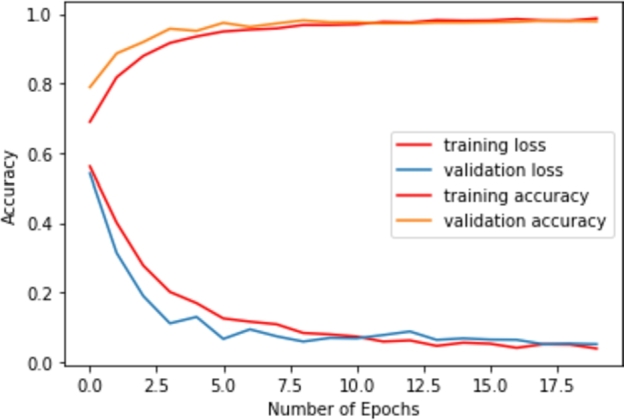
The training learning curve of the CNN architecture.

Face mask detection uses a customized convolutional neural network in this work. The training of the network was conducted using 20 epochs. Fig. 21 shows the training learning curve of the architecture. The rise in the accuracy of both the train and validation set is observed in the curves. The train-validation-test split was 80:10:10, respectively. So, the test size was 10%. The authors decided to run 18 epochs for the training. The average epoch time was 143 seconds, where the maximum epoch time was 145 seconds, and the lowest was 140 seconds. After ten epochs, the validation loss began to rise. The validation loss was the training monitor. As a result, the training was halted. The best outcome in terms of accuracy and loss trade-offs between train sets and validation sets was Model 10, which was chosen.

Table 4 shows the accuracy of the CNN model for train, validation, and test set. According to this table, the test accuracy of the proposed CNN architecture is 97.90%.

**Table 4:** Model accuracy and loss for train, test, and validation set.

Sets	Train	Dev	Test
Accuracy	98.69%	98.57%	97.90%
Loss	3.83	3.61	12.18

**Figure 22:**
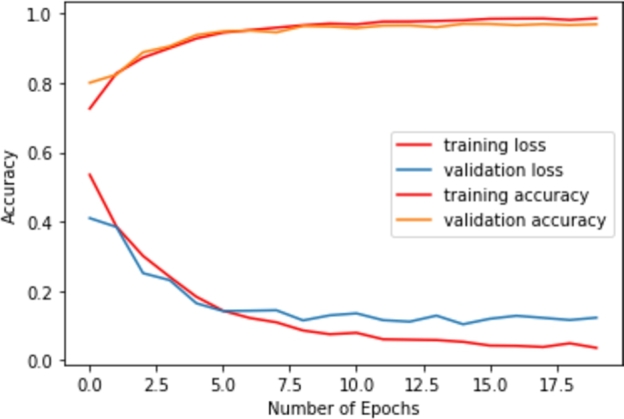
Training and testing curves for domain adaption technique.

Next, the domain adaption technique is applied to evaluate the performance of the face mask detection model, where different sources of datasets are employed for training and testing the proposed system. The customized CNN model has been trained by the source dataset [32] and finally, it has been evaluated by the target images of [33]. Training and testing accuracy and loss with the change of epochs for domain adaption technique have been depicted in Fig. 22.

**Figure 23:**
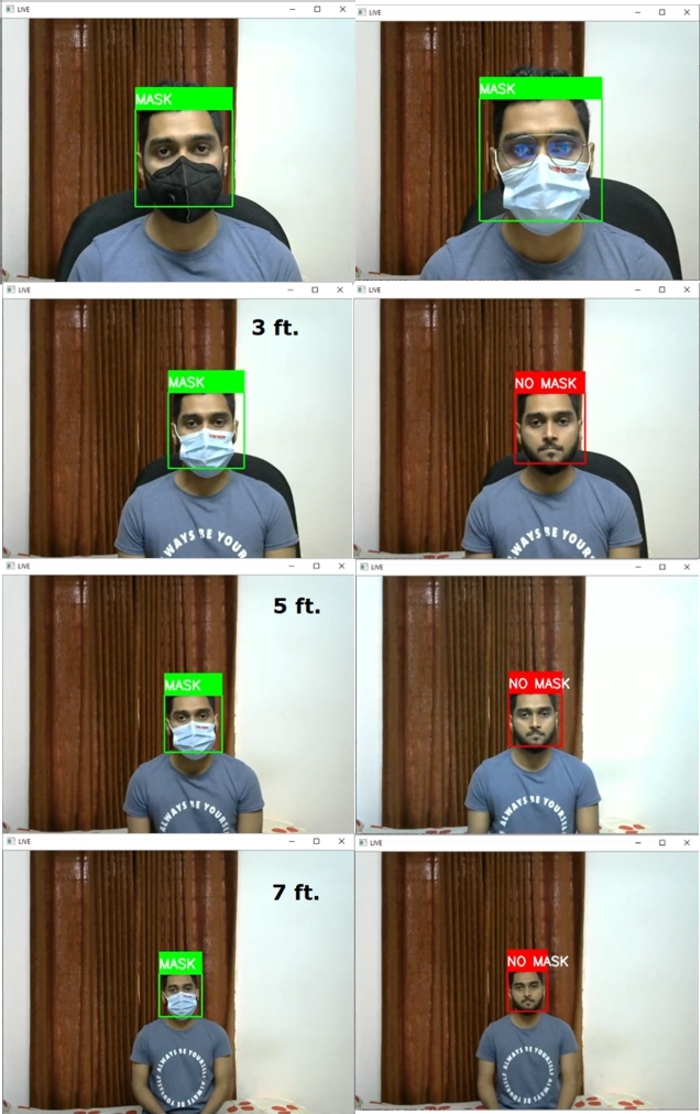
Successful predictions of face mask detection model in varying distance and with or without eyeglass.

Fig. 23 shows the trained face mask detection model's successful predictions at varying distances. Various masks were evaluated, and the model successfully predicted them. The model was tested from 2 ft. to 7 ft. distance range and successfully predicted the face mask. The system can detect a wide variety of face masks. Even if the person wears eyeglasses, it can detect the face mask.

**Figure 24:**
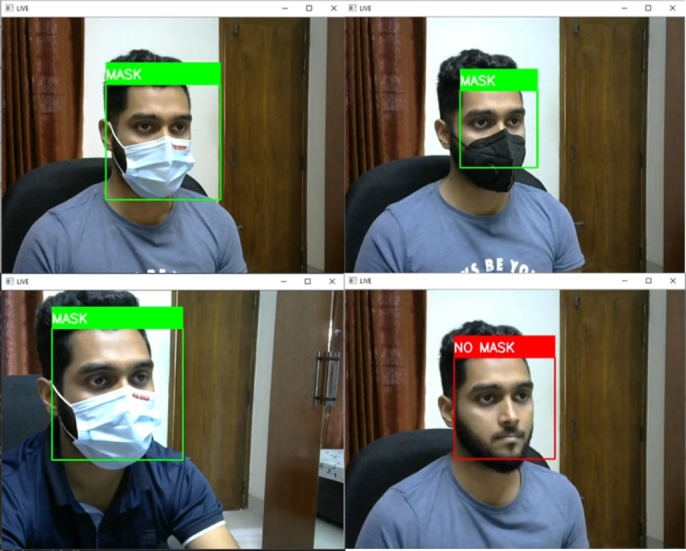
Successful predictions of face mask detection model in oblique projection.

The application of this system demands prediction in oblique projection. So, an oblique projection test was performed, and the model showed satisfactory results in these assessments. Fig. 24 shows the successful prediction of the face mask model in oblique projection.

**Figure 25:**
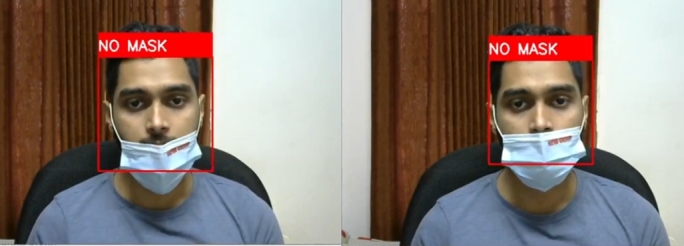
Successful predictions of wrongly worn face mask.

This system is capable of detecting wrongly worn face masks. Fig. 25 shows wrongly worn face mask is being classified as no mask scenarios.

**Figure 26:**
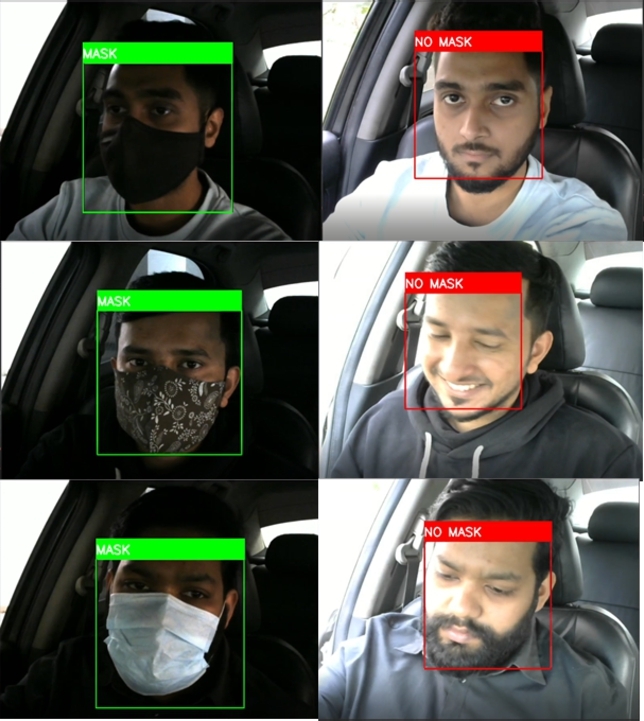
Successful predictions of face masks in real-life scenarios.

The face mask model was called into question in a variety of scenarios. The system was sorely tested in a vehicle by the volunteers. Fig. 26 depicts the successful identification of a face mask in a real-world experiment. Here the experiments were performed in varying lighting conditions.

**Figure 27:**
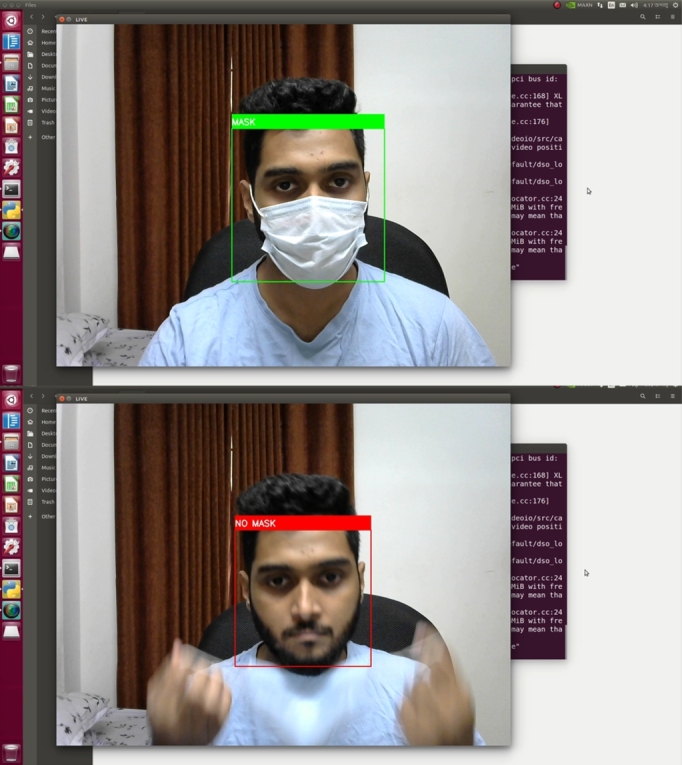
Successful predictions of face mask with Nvidia Jetson Nano.

The primary usable system is fully embedded, and the Nvidia Jetson Nano deploys the computer vision module in this research. Next, the face mask model was also deployed in Nvidia Jetson Nano. Fig. 27 shows the successful detection of the face mask with the Nvidia Jetson Nano developer kit.

#### 4.1.2 Fatigue and sleep detection experiments and evaluations

**Figure 28:**
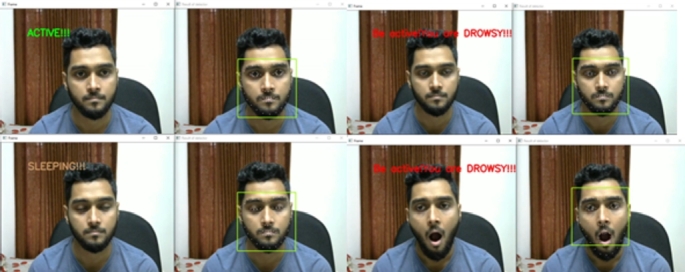
States classification from a regular distance.

The system can detect different types of face masks. Even if the person is wearing eyeglasses, it can identify the face mask. The system is designed to classify the three states of a driver. They are the active state, the fatigue state, and the sleeping state. Yawn detection is also classified as fatigue state detection. The drowsiness detection system has passed through several experiments with varying lighting conditions, distances, and projections. These experiments were performed on a Windows desktop computer and an Nvidia Jetson Nano, similar to face mask detection. Some real-life scenarios were experimented with by the volunteers. Fig. 28 shows the state's classification of the computer vision module. The system can successfully classify all three states.

**Figure 29:**
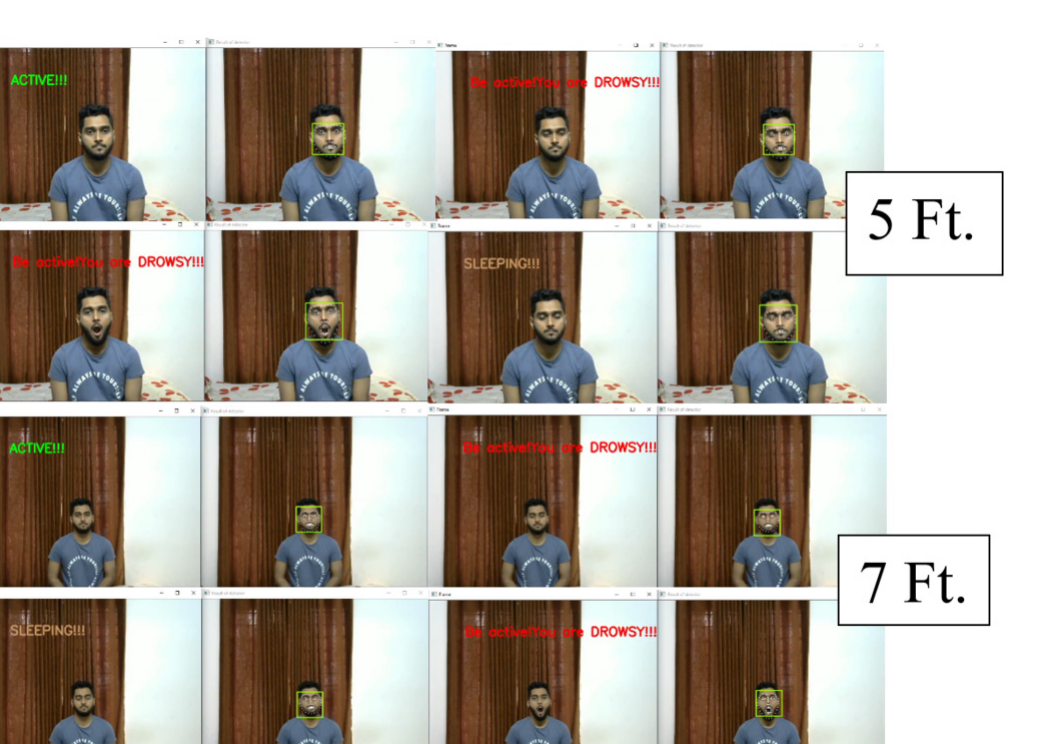
States classification from varying distances.

The same experiment was carried out for varying distances, just as the face mask model was assessed. Fig. 29 shows the experimental results where the distance varied from 3 feet to 7 feet. The results were satisfying and the system classified all the states accurately.

**Figure 30:**
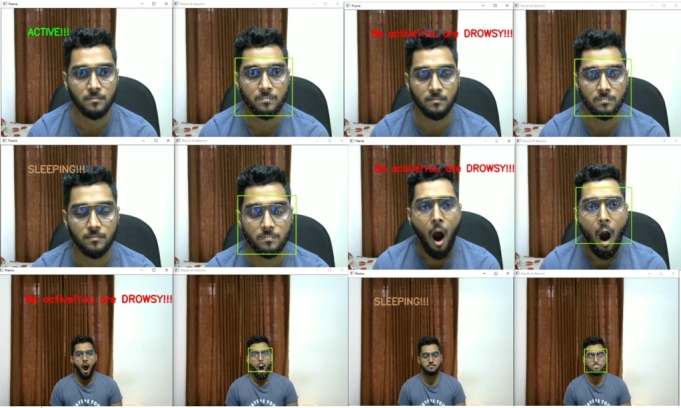
States classification from varying distances with eyeglasses.

This system can classify all the states accurately, even if the person is wearing eyeglasses. At various distances, an experiment with eyeglasses was conducted. The system successfully classified all the states from even a 7-foot distance with the eyeglasses. Fig. 30 shows the results from an eyeglass test for varying distances.

**Figure 31:**
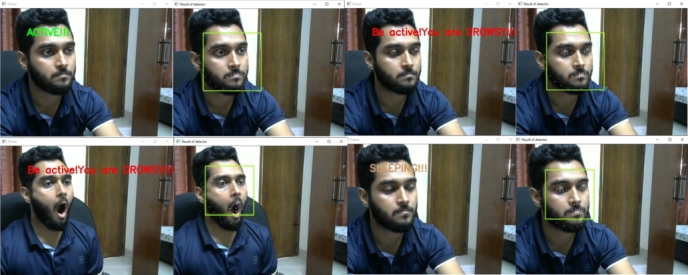
States classification on oblique view projection.

As the application demands oblique projection, this state's classification was tested on oblique view projection. Fig. 31 shows the state's classification on oblique view projection.

**Figure 32:**
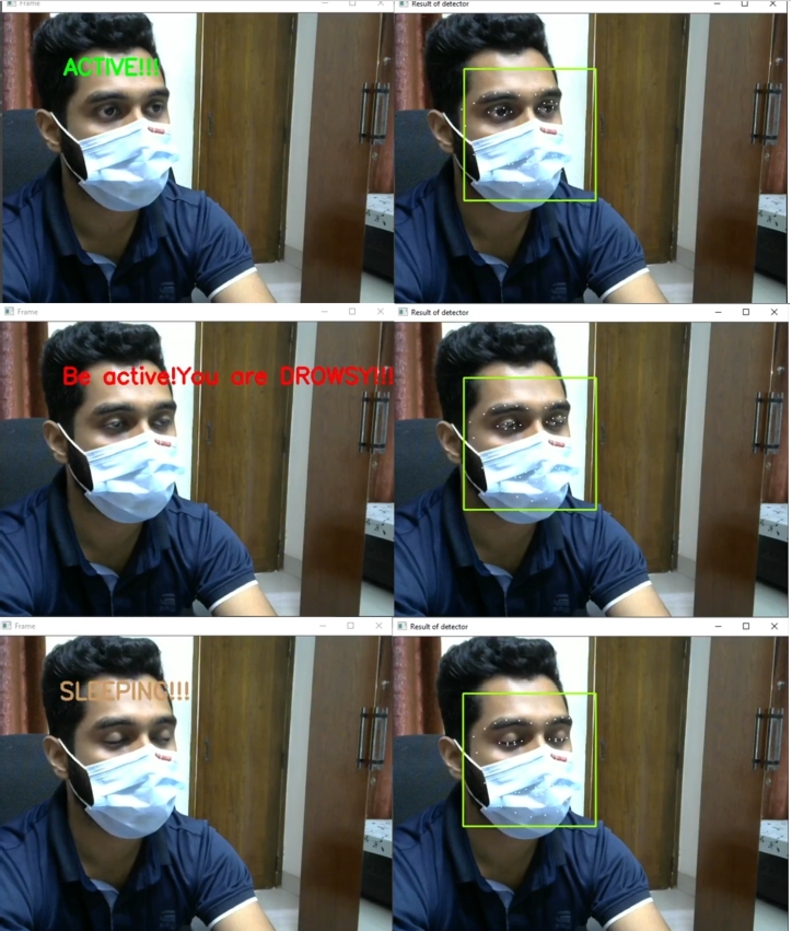
States classification with the face mask in oblique projection.

The system can detect all three states only except the yawn detection while wearing a face mask. This capability makes the system more rigid and reliable to perform in such situations. Fig. 32 shows the result of states classification with the face mask.

**Figure 33:**
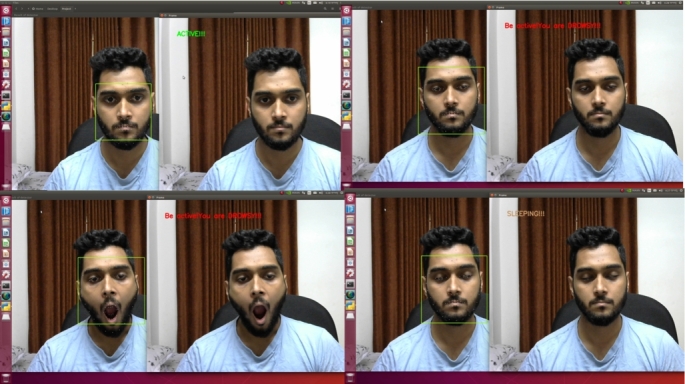
States classification from the Nvidia Jetson Nano.

Next, the embedded system test on Nvidia Jetson Nano was performed for states classification. The Nvidia Jetson Nano's state classification results are shown in Fig. 33.

**Figure 34:**
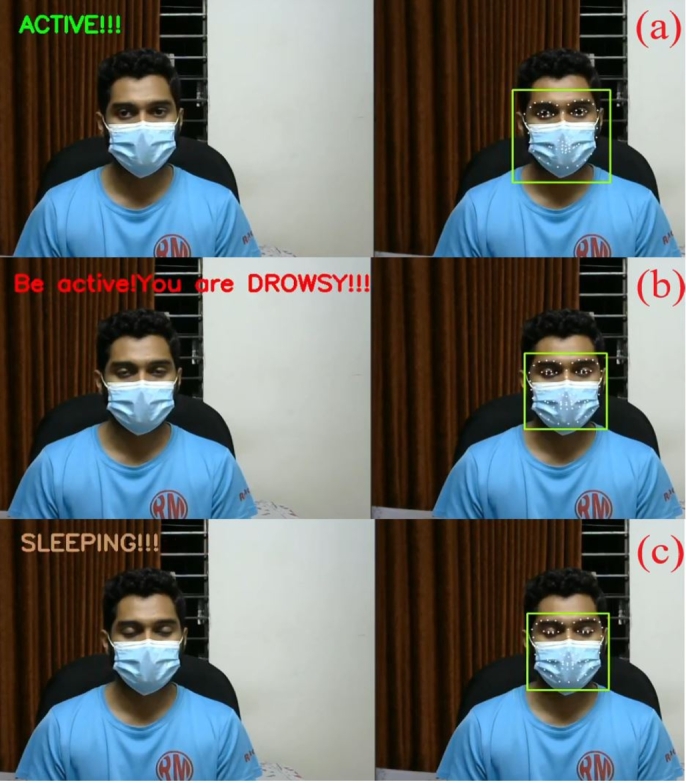
States classification from 5 feet distance with wearing face masks; (a) Active, (b) Drowsy and (c) Sleeping states.

In this work, the proposed system detects driver drowsiness with the heart rate module and EAR ratio (eye openness or closeness) using eye landmark points while wearing face masks. On the other hand, the proposed system detects driver drowsiness with the heart rate module, EAR ratio (eye openness or closeness) and yawning using eye and lip landmark points without face masks. Fig. 34 illustrates various state classifications of the proposed system from 5 feet distance with wearing face masks in the lab experiments.

**Figure 35:**
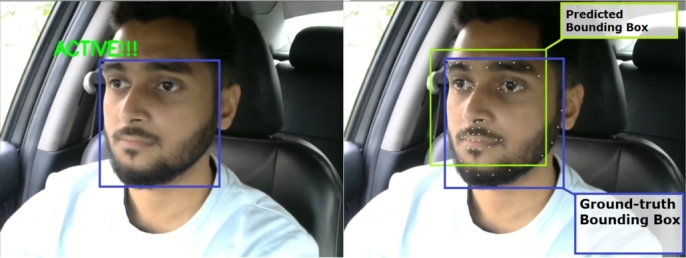
Predicted bounding box vs. the ground-truth bounding box.

Next, four volunteers (three male and one female) conducted real-life scenario experiments with the proposed system in a sedan and an SUV car. The car had been driven over the four distinct routes in the Bashundhara Residential Area of Dhaka, Bangladesh. The anticipated face bounding boxes were compared to the ground-truth bounding boxes. There is no significant disparity between the ground-truth and predicted bounding boxes. When compared to the ground-truth results, the results were also accurate. Fig. 35 exhibits the ground-truth bounding box in blue and the predicted bounding box in green.

**Figure 36:**
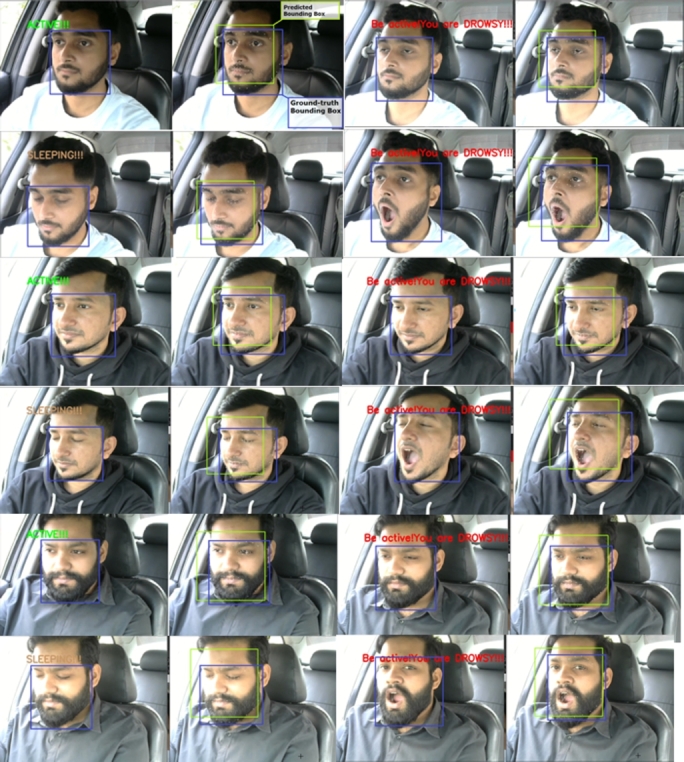
In-vehicle states classification with predicted and ground-truth bounding boxes.

Fig. 36 shows the real-life states classification results. All the states were classified accurately, and the predicted bounding box was close to the ground-truth bounding box.

All the experimental data was inscribed, and then the performance was evaluated. Table 5 shows the system performance in states classification for varying lighting conditions. Here, a three-light set-up was used to illustrate the full light conditions from the front. Two of the three lights represented a moderate lighting case, while only the third light represented a low lighting condition.

**Table 5:** System performance evaluation on states classification.

	Active Ground State	True classification	Active accuracy	Fatigue Ground State	True classification	Fatigue accuracy	Sleep Ground State	True classification	Sleep accuracy
Full light	200	191	95.5%	200	188	94%	200	195	97.5%
Moderate light	200	194	97%	200	195	97.5%	200	169	84.5%
Low light	200	172	86%	200	163	81.5%	200	180	90%

**Figure 37:**
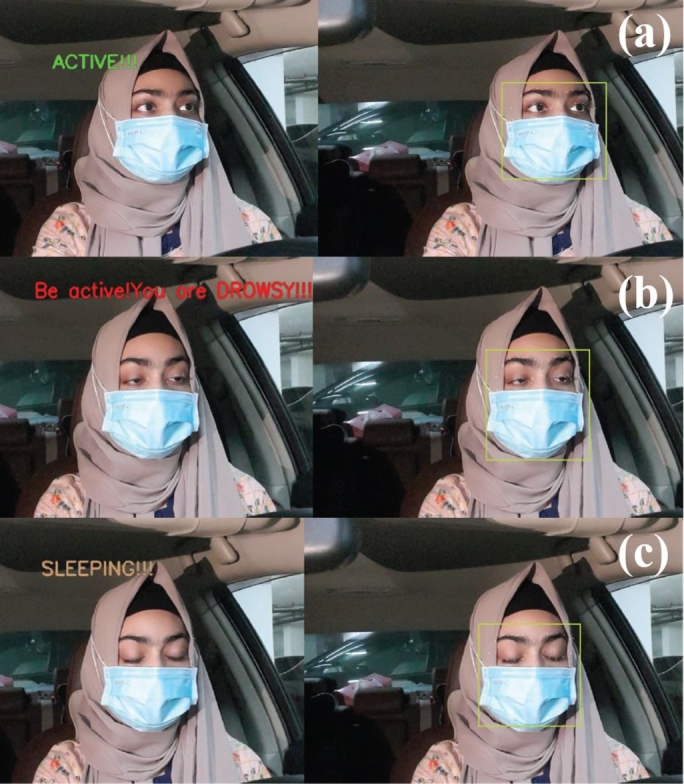
States classification for real-life experiments with wearing face masks; (a) Active, (b) Drowsy and (c) Sleeping states.

As shown in Fig. 37, the proposed system identifies various states of driver fatigue with the heart rate module and EAR ratio (eye openness or closeness) using eye landmark points while wearing face masks for real-life experiments in an SUV car.

### 4.2 Experiments and evaluation of heart rate module

The heart rate module designed in this work was tested and evaluated by eight volunteers, where two of them were females, and six of them were males. Each volunteer evaluated the heart rate module for up to 4 minutes. The ages of the volunteers were between 22 to 55 years. The produced results were assessed and compared with two industry-standard heart rate measuring devices. The overall results were satisfactory. The results sometimes fluctuated due to unusual electrodes placement.

**Figure 38:**
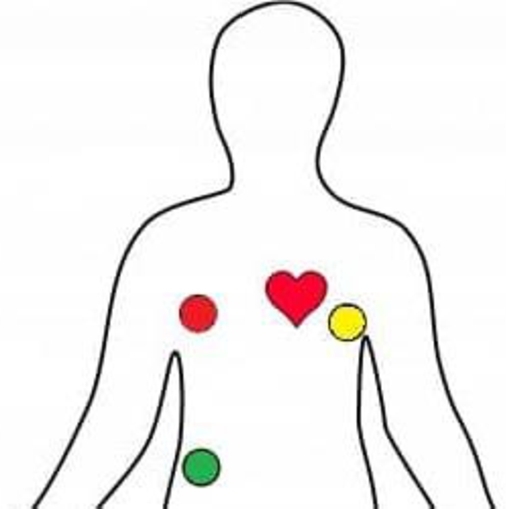
The employed zone in the chest under the garments to attach the electrodes for heart rate module.

The authors attempted to implant the electrodes in various body locations, e.g., fingertips, arms, and chest. Finally, the authors placed the electrodes to get the best output during the lab experiments and real-time drive tests, as illustrated in Fig. 38. The chest hairs were clipped off and the three leads were implanted below the left and right clavicles and lower abdomen chest regions under the garments of the users. It was difficult for the volunteers to demonstrate such fatigue states where the heart rate drops under the BPM threshold. The volunteers could achieve that, but that stayed for a bit of time. To prove the system's accuracy, the authors tested the heart rate module with a real-time comparison between Huawei Watch GT-2 and the OMRON Automatic Blood Pressure Monitor HEM-7120. Both of them are industry-standard commercial devices to get the BPM.

Table 6 shows the performance evaluation based on existing industry standard machines. The results were satisfactory considering the pricing. Total 50 BPM values were compared in this work. According to this table, the proposed device's root-mean-square deviations (RMSD) with the Omron machine and Huawei watch are 2.42 and 2.59, respectively.

**Table 6:** Root-mean-square deviation of the proposed device with commercial machines in BPM.

	Omron machine	Huawei Watch
RMSD (BPM)	2.42	2.59

### 4.3 Overall performance evaluation

The overall performance of the complete embedded system is satisfactory. Table 7 shows the comparison of this system with existing similar works. According to Table 7, very few automatic driver drowsiness detection system uses the edge computing device Jetson Nano. The proposed device outperformed most of the other similar devices in terms of behavioral and physiological measurement, maximum distance covered, working with face mask and eyeglasses, deployment on an embedded platform, accuracy, etc.

**Table 7:** System comparison with existing similar systems.

Works Compared	Method	Behavioral and Physiological measure	Max distance covered	Works with eyeglass?	Face mask?	Heart rate sensor?	Alarm	Full embedded?	Accuracy
[5]	SWM	No	Not mentioned	Not mentioned	No	No	No	No	Low
[6]	Computer Vision	Yes	2 ft. to 3 ft.	Not mentioned	No	No	No	No	Moderate
[11]	RNN, and CNN	Only behavioral	Not mentioned	Yes	No	No	Yes	Smartphone application	High
[12]	CNN, and Computer vision	Only behavioral	Not mentioned	Yes	No	No	Yes	Nvidia Jetson Nano	Moderate
[13]	Computer vision	Only behavioral	Not mentioned	Yes	No	No	Yes	Raspberry Pi3 model B	High
[14]	Computer vision	Only behavioral	Not mentioned	Not mentioned	No	No	Yes	Android smartphone application	High
[15]	RNN, Computer vision, EEG, and gyroscope	Yes	Not mentioned	Not mentioned	No	No	Yes	Yes	High
[35]	Wireless wearable device	Yes	Not mentioned	Not mentioned	No	Yes	No	No	Moderate
This system	CNN, Computer vision, and HRV	Yes	7 feet	Yes	Yes	Yes	Yes	Nvidia Jetson Nano	High

**Figure 39:**
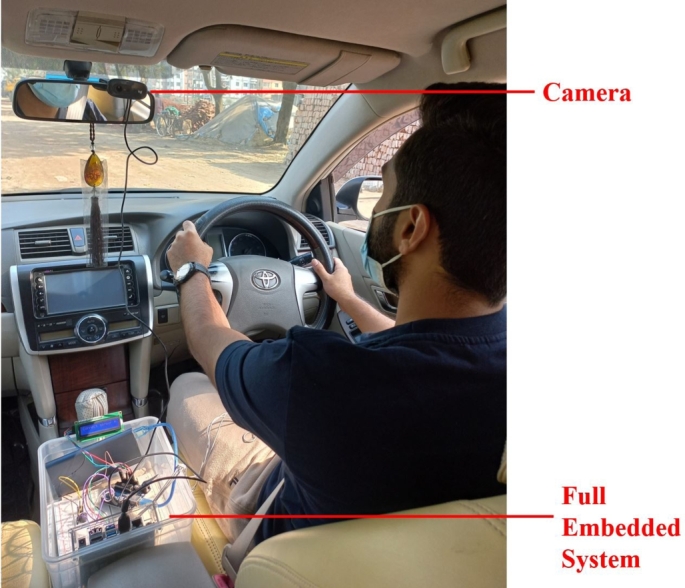
Illustration of full embedded setup of the proposed driver drowsiness detection system.

This method has several strong qualities that make it distinctive and dependable. The entire system is contained within a single box. The whole setup of this embedded system is shown in Fig. 39. The Nvidia Jetson Nano developer kit powers the computer vision module of this system. The utilization of the Nvidia Jetson Nano adds to the system's uniqueness and reliability.

The critical system ran so well thanks to this powerful piece of technology. It can detect any signs of fatigue from a sufficient distance of 7 feet, which is more than enough for almost every vehicle. This work has been tested and found to detect fatigue while the driver is wearing the glass successfully. In this pandemic condition, the addition of face mask detection made this system stand out. When compared to other existing systems, the system's performance appeared promising.

## 5 Conclusion and future works

This paper develops an automatic driver's fatigue detection system through computer vision with eye movement tracking, yawn detection, and heart rate monitoring. The eye aspect ratio (EAR) and lip distance computation are applied for the driver's various states classification, e.g., active, drowsy, and sleepy. The onboard Arduino Uno and AD8232 heart rate module have also been used, which constantly measures the heart rates and detects any fluctuation in BPM related to the drowsiness. Next, a convolutional neural network-based face mask detection framework has been developed to tackle the current pandemic. Finally, the proposed three techniques are integrated into one system for driver sleepiness detection. The performance of the implemented prediction framework has been tested on a desktop Windows PC with various simulated and real-life scenarios such as oblique projection, different lighting conditions, changing positions of the device from its user, with and without eyeglasses, etc. Finally, the proposed drowsiness technique has been deployed in an edge computing device, Nvidia Jetson Nano. This portable and user-friendly device can assist drivers in reducing road accidents as well as the pain and anguish that individuals experience in their daily lives. The designed prototype is a low-cost alternative to other proposed ways for detecting fatigue, and the entire system can easily be integrated into a variety of vehicles. Because of its portability and low cost, the device could be a key differentiator in reducing traffic accidents in developing countries like Bangladesh due to human error.

The authors plan to use a custom-built neural network to construct the entire detection mechanism in the future. The authors aim to create a dataset for all of the states, both with and without the face mask. The entire system will be more compact and faster by employing a single model. Drowsiness detection by brainwaves [45] is another reliable physiological measure that the authors want to introduce. This implementation will aid in the faster and more accurate detection of fatigue levels. Another future goal is to detect fatigue levels in absolutely dark environments using a night vision camera module. The frames will be put to the test using a CNN model that will be trained on night vision image samples. A future extension of this work is to use smart textile-woven ECG electrodes.

## Declarations

### Author contribution statement

Ashiqur Rahman: Conceived and designed the experiments; Performed the experiments.

Mamun Bin Harun Hriday: Contributed reagents, materials, analysis tools or data; Wrote the paper.

Riasat Khan: Analyzed and interpreted the data; Wrote the paper.

### Funding statement

This research did not receive any specific grant from funding agencies in the public, commercial, or not-for-profit sectors.

### Data availability statement

Data included in article/supp. material/referenced in article.

### Declaration of interests statement

The authors declare no conflict of interest.

### Additional information

No additional information is available for this paper.
